# Organelle-Specific Nitric Oxide Detection in Living Cells via HaloTag Protein Labeling

**DOI:** 10.1371/journal.pone.0123986

**Published:** 2015-04-29

**Authors:** Jianhua Wang, Yuzheng Zhao, Chao Wang, Qian Zhu, Zengmin Du, Aiguo Hu, Yi Yang

**Affiliations:** 1 Synthetic Biology and Biotechnology Laboratory, State Key Laboratory of Bioreactor Engineering, CAS Center for Excellence in Brain Science, School of Pharmacy, East China University of Science and Technology, 130 Mei Long Road, Shanghai, 200237, P. R. China; 2 Shanghai Key Laboratory of Advanced Polymeric Materials, School of Materials Science and Engineering, East China University of Science and Technology, 130 Mei Long Road, Shanghai, 200237, P. R. China; University Francisco de Vitoria School of Medicine, SPAIN

## Abstract

Nitric oxide (NO) is a membrane-permeable signaling molecule that is constantly produced, transferred, and consumed *in vivo*. NO participates and plays important roles in multiple biological processes. However, spatiotemporal imaging of NO in living cells is challenging. To fill the gap in currently used techniques, we exploited the versatility of HaloTag technology and synthesized a novel organelle-targetable fluorescent probe called HTDAF-2DA. We demonstrate the utility of the probe by monitoring subcellular NO dynamics. The developed strategy enables precise determination of local NO function.

## Introduction

Nitric oxide (NO), a ubiquitous and uncharged free radical, is an intracellular and intercellular messenger that governs a myriad of physiological and pathophysiological processes, including vascular homeostasis, neurotransmission, immune systems, and tumor progression [1–3]. Various methods can be used to quantify NO, such as colorimetric [4], fluorometric [5, 6], electrochemical [7], electron paramagnetic resonance spectroscopy [8], and chemiluminescence [9] techniques.

Fluorescence labeling and imaging have become the most promising techniques for NO sensing because of their selectivity, sensitivity, and spatiotemporal resolution. Well-developed, small-molecule-based fluorescent probes for NO have beenpromoted, including diaminoaromatic fluorescent compounds [10–12] and copper–fluorescein complex [6]. These probes respond specifically, rapidly, and directly to NO at low concentrations, as well as allow NO visualization in single cell. However, none of them can be used to monitor the subcellular distribution of NO, which depends on membrane permeability and NO reactivity, as well as the reduction–oxidation states of intracellular compartments. In our previous study, we observed that protein S-nitrosation derived from endogenous NO production mainly exists in the mitochondria and peri-mitochondrial compartment [13].

To further elucidate the complex biological functions of NO, an ideal NO probe should be able to report the local and subcellular changes in NO concentration. HaloTag is a specific and covalent protein-labeling technology that employs alkyl chloride as reactive moiety, which can covalently bind to a modified bacterial haloalkane dehalogenase (HaloTag) [14, 15]. Various HaloTag ligands have been developed for targeted protein labeling [15], protein immobilization [16], super-resolution imaging [17], and magnetic resonance imaging [18]. Based on this specific protein–ligand interaction, we synthesized a novel organelle-targetable fluorescent probe, HTDAF-2DA, which could be used for real-time imaging of NO with fine temporal and spatial resolutions in living cells.

## Materials and Methods

### Chemicals and reagents

All solvents were of analytical grade and used after appropriate distillation or purification. Other reagents were commercial chemicals and used as received. All aqueous solutions were prepared with Millipore water. 6-Carboxyfluorescein and 2-[2-(6-chloro-hexyloxy)-ethoxy]-ethylammonium hydrochloride were synthesized in accordance to the literature [15, 19]. DAF-2DA was prepared in accordance to the literature with minor modifications [10]. Dimethyl sulfoxide, 4’,6diamidino-2-phenylindole (DAPI), Lipopolysaccharides (LPS), Nω-Nitro-L-arginine methyl ester hydrochloride (L-NAME), xanthine and xanthine oxidase were obtained from Sigma. Recombinant Murine interferon-γ (IFN-γ) was obtained from Peprotech. MitoTracker Red FM was from Invitrogen. DEA NONOate and MAHMA NONOate were purchased from Cayman Chemical Co. Dulbecco’s phosphate-buffered saline (PBS) powder was from Hyclone. Dulbecco’s modified Eagle’s medium (DMEM) and fetal bovine serum (FBS) were from Gibco.

### Synthetic routes and details of HTDAF-2DA

2-Bromo-*N*-{2-[2-(6-chloro-hexyloxy)-ethoxy]-ethyl}-acetamide (595 mg, 1.73 mmol) was added to a solution of DAF-2DA (847 mg, 1.90 mmol), anhydrous potassium carbonate (394 mg, 2.85 mmol), and sodium iodide (52 mg, 3.47 mmol) in *N*,*N*-dimethylformamide (7 ml). The mixture was stirred under nitrogen at room temperature for 24 h. The solvent was removed in vacuo at a low temperature. The residue was then purified by column chromatography over silica gel and subjected to preparative high-performance liquid chromatography purification. The solvent was removed in vacuo at a low temperature to yield HTDAF-2DA (55 mg, 6.1%), with a recovery of DAF-2DA (0.45 g). Hydrogen-1 nuclear magnetic resonance (NMR) (CDCl_3_) δ: 1.40 (m, 4H, -CH_2_-), 1.60 (m, 2H, -CH_2_-), 1.75 (m, 2H, -CH_2_-), 2.31 (s, 6H, COCH_3_), 3.40–3.70 (m, 14H, CH_2_-N, CH_2_-O, CH_2_-Cl), 6.33 (s, 1H, ArH), 6.80 (dd, 2H, ArH, *J* = 8.8, 2.0 Hz), 6.93 (d, 2H, ArH, *J* = 8.8 Hz), 6.98 (s, 1H, NH), 7.03 (d, 2H, ArH, *J* = 2.0 Hz), 7.26 (s, 1H, ArH) ppm. Carbon-13 NMR (CDCl_3_) δ: 21.2, 25.3, 26.7, 29.1, 29.3, 29.7, 32.5, 45.1, 69.7, 69.9, 70.0, 71.3, 110.2, 110.4, 110.6, 117.4, 117.6, 117.8, 118.1, 128.9, 129.2, 151.5, 151.7, 152.0, 152.2, 169.0, 169.1, 169.2. High-resolution mass-spectrometry: m/z calculated for M + H 710.2480, found 710.2482.

### Plasmid construction

To construct a vector for HaloTag expression in mammalian cells, the HaloTag gene was amplified through polymerase chain reaction and then cloned into pIRESneo2 vector with non-tagged sequences for cytosolic expression [20–23]. For nuclear targeting, threefold nuclear localization signal (3×NLS) DPKKKRKVDPKKKRKVDPKKKRKV was added to the C-terminus [24–26]. The molecular mass of Nuc-HaloTag ptrotein was 37.03 kDa. For plasma membrane targeting, the plasma membrane localization signal MLCCMRRTKQVEKNDEDQKI was inserted at the N-terminus [27–29], and a linker (SELKLRILQSTVPRARDPPVATM) existed between the localization signal and HaloTag; thus, the molecular mass of Mem-HaloTag protein was 38.56 kDa. The mito-HaloTag expression vector was constructed by fusing a duplicated mitochondrial targeting signal MSVLTPLLLRGLTGSARRLPVPRA KIHSLGDLSVLTPLLLRGLTGSARRLPVPRAKIHSLGD at the N-terminus [30–33], and cloned into pcDNA3.1-Hygro(+) (Invitrogen) vector. The molecular mass of Mit-HaloTag protein was 40.10 kDa. The mCherry gene was cloned into BamHI/HindIII sites of pcDNA3.1-Hygro(+)(invitrogen) vector with non-tagged sequences for cytosolic expression. A duplicated mitochondrial targeting signal was cloned into BamHI/HindIII sites, yielding pcDNA3.1-Mito-mCherry. The plasma membrane localization signal was cloned into BamHI/HindIII sites, yielding pcDNA3.1-membrane-mCherry. 3×NLS was cloned into BamHI/HindIII sites, yielding pcDNA3.1-nuclear-mCherry.

### Characterization of HTDAF-2 in vitro

HTDAF-2 was stored at –20°C in the dark until analysis. The stock solutions of NO donor and HTDAF-2 were diluted with PBS (pH 7.4). Fluorescence spectra were measured by a Cary Eclipse spectrofluorometer (Varian). For excitation scans, the emission wavelength was set to 525 nm while scanning the excitation spectra at 1 nm increments from 400 nm to 500 nm. For emission scans, the excitation wavelength was set to 480 nm while scanning the emission spectra at 1 nm increments from 500 nm to 600 nm. The fluorescence response of HTDAF-2 to NO donor (or other chemicals) at varied concentrations was measured by a Synergy 2 Multi-mode Microplate Reader with excitation filter 485 BP 20 nm and emission filter 528 BP 20 nm (BioTek). Each assay was performed with 25 μL of compounds and 50 μL of HTDAF-2 in a 384-well flat-bottom microplate (Greiner). Fluorescence intensity was measured immediately.

### Cell culture

HeLa and MCF-7 cells were grown in DMEM with 10% FBS at 37°C in a humidified atmosphere of 95% air and 5% CO_2_. Raw 264.7 Macrophage cells were maintained in RPMI-1640 with 10% FBS. Cells were plated in antibiotic-free medium supplemented with 10% FBS 16 h before transfection. Cells were transfected using FuGene HD transfection reagent (Promega) in accordance to the methods of the manufacturer.

### Fluorescence detection of HaloTag labeled with HTDAF-2 using sodium dodecyl sulfate (SDS)-polyacrylamide gel electrophoresis (PAGE)

The reactions between HaloTag protein and HTDAF-2 were verified by 15% SDS-PAGE. Cells expressing HaloTag proteins and labeled with HTDAF-2DA were solubilized in SDS gel loading buffer (2% SDS, 10% glycerol, 0.01% bromophenol blue,) and boiled for 5 min. Electrophoresis was performed in the dark. The gel was imaged by a Carestream In-Vivo Imaging FX System (Excitation: 490/20 nm, Emission: 535/50 nm).

### Live-cell fluorescence measurement using a microplate reader

HeLa and MCF-7 cells were incubated at 37°C in DMEM containing 5 μM HTDAF-2DA for loading 36 h after transfection. After 15 min, cells were rinsed four times with PBS and twice with DMEM, as well as incubated in DMEM for 1 h. During incubation, the medium was replaced every 20 min. The medium was then replaced with PBS or PBS with NO donor, and the cells were used for detection. Raw 264.7 cells were stimulated for 8 h with LPS (0.5 μg/ml) plus IFN-γ (250 U/ml) with or without L-NAME (2 mM) 24 h after transfection. Cells were labelled with 5 μM HTDAF-2DA for 30 min and rinsed six times with medium, as well as incubated in RPMI 1640 medium for 1 h. Fluorescence excitation at 485 nm was measured by a Synergy 2 Multi-mode Microplate Reader with excitation filter 485 BP 20 nm and emission filter 528 BP 20 nm. Fluorescence values were background-corrected by subtracting the intensity of HeLa cells that did not express HaloTag protein.

### Imaging of HaloTag protein labeled with HTDAF-2DA in living cells

HeLa cells were placed on 35 mm glass-bottom culture dishes (NEST Biotechnology Co. Ltd.) in DMEM supplemented with 10% (v/v) FBS, and observed 36 h post-transfection. HaloTag proteins were expressed in different subcellular compartments by tagging with organelle-specific signal peptides. Cells were labeled in accordance to our previously described procedure. For HTDAF-2DA co-labeling with nuclear dye DAPI, cells were first labeled by 5 μM HTDAF-2DA as described and then by 75 μM DAPI for 30 min at 37°C. For co-labeling of HTDAF-2DA, DAPI and MitoTracker Red FM, cells were first labeled by 5 μM HTDAF-2DA as described and then by 75 μM DAPI and 300 nM MitoTracker Red FM. Images were acquired using a high-performance fluorescent microscopy system equipped with a Nikon Eclipse Ti-E automatic microscope, a cooled monochrome digital camera head DS-Qi1 Mc-U2, and a highly stable Shutter Lambda XL light source. A Plan Apo 40×0.95 NA objective was used. For imaging, 482 BP 35 nm band-pass excitation filter and 535/40 emission filter altered by a Lambda 10-XL filter wheel (Shutter Instruments) were used. Images were captured in 640 × 480 format, 12 bit depth, and 60 ms exposure for the channel.

### Statistical analysis

Data are presented either as a representative example of a single experiment repeated at least in triplicate or as three or more experiments. Data obtained are represented as mean values ± SD. All P values were obtained using unpaired two-tailed Student’s t-test. Values of p<0.05 were considered statistically significant (*0.01 < p < 0.05; **0.001 < p < 0.01; ***p < 0.001).

## Results and Discussions

### Design and synthesis of HTDAF-2DA

**Fig 1:**
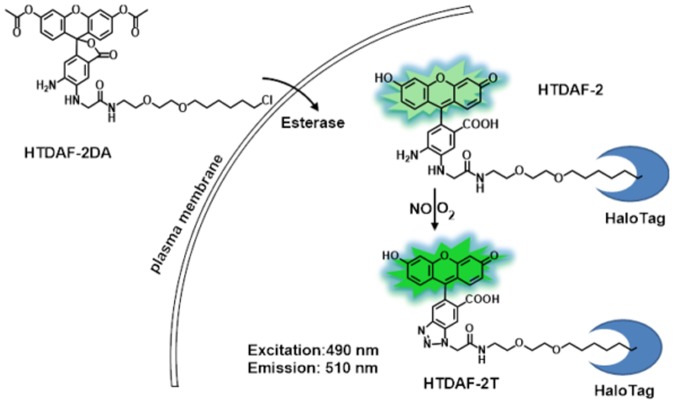
Structures and reaction chemistry of HTDAF-2DA.

We designed and synthesized HTDAF-2DA for the selective detection of NO. HTDAF-2DA was synthesized in three steps (Scheme A in S1 File). The probe was a monoalkylated derivative of DAF-2DA, a small-molecule fluorescent NO probe widely used for detecting and imaging NO in living cells [10]. Similar to DAF-2 DA [34], the diacetate groups of HTDAF-2DA can be hydrolyzed by esterases after they permeate the cells. The produced HTDAF-2 can then be immobilized by the HaloTag protein, which can be targeted into different subcellular compartments [15] and reacted with NO under aerobic conditions to form a triazole product (Fig 1). This product suppresses the photo-induced electron transfer process and activates the fluorescence of the probe. Another compound, HTFAM, was synthesized and used as a control (Scheme B in S1 File). HTFAM has a structure similar to that of HTDAF-2 but does not react with NO.

### Characterization of HTDAF-2DA

**Fig 2:**
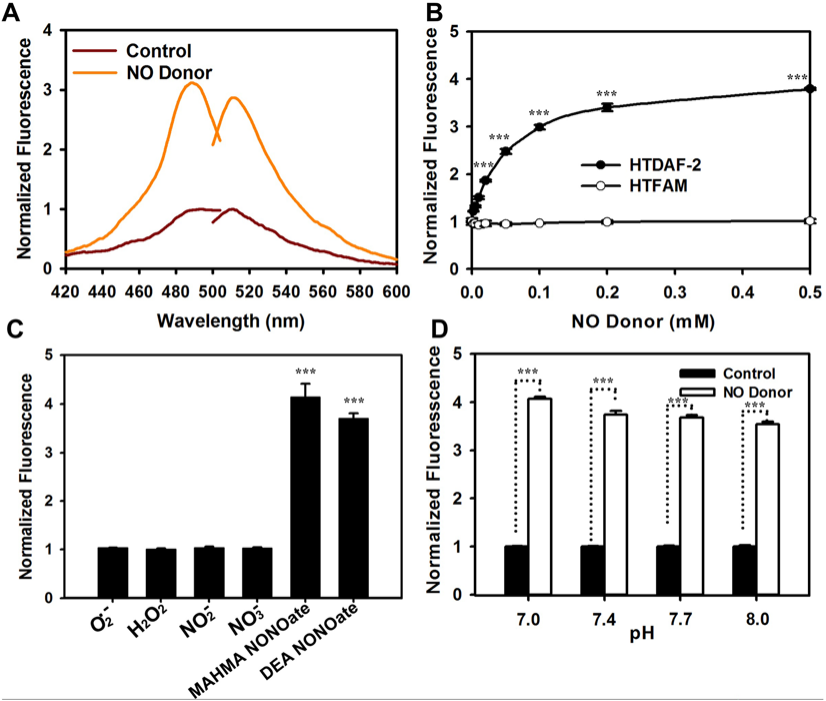
Properties of NO Sensor HTDAF-2. (A) Fluorescence spectra of HTDAF-2. Fluorescence excitation and emission spectra of 10 nM HTDAF-2 in PBS (pH 7.4) before (dark red lines) and after (orange lines) the addition of 0.5 mM NO donor (DEA NONOate) at 25°C. Excitation spectrum recorded at an emission wavelength of 525 nm shows a maximum at 488 nm. Emission spectrum recorded at an excitation wavelength of 480 nm shows a maximum at 512 nm. (B) The fluorescence intensities of HTDAF-2 in the presence of different concentrations of NO donor (DEA NONOate) normalized to the initial value. (C) The fluorescence response of HTDAF-2 after the addition of 2 mM xanthine/20 mU xanthine oxidase, 0.5 mM H_2_O_2_, NO^2−^, NO^3−^, MAHMA NONOate, and DEA NONOate for 30 min in PBS solution. (D) The fluorescence response of HTDAF-2 to NO donor (DEA NONOate) at the indicated pH. Error bars represent the standard deviation (SD).

In vitro studies showed that HTDAF-2DA exhibited weak fluorescence. However, when the diacetate groups were hydrolyzed in the presence of 1 N NaOH, the fluorescence markedly increased (S1A Fig). HTDAF-2 displayed maximum fluorescence excitation and emission at 490 and 510 nm (Fig 2A), which substantially increased upon the addition of NO donor (MAHMA NONOate and DEA NONOate) (Fig 2B and 2C). In the control, the fluorescence of HTFAM did not respond to the NO donor (Fig 2B). The fluorescence intensity of HTDAF-2 did not respond noticeably to other reactive oxygen species and reactive nitrogen species, such as O_2_^−^, H_2_O_2_, NO^2−^, and NO^3−^ (Fig 2C). These results indicated that HTDAF-2 was a highly specific fluorescent probe for NO.

The fluorescence of HTDAF-2 and its product after reacting with NO was insignificantly sensitive to pH 7.0 to 8.0 (Fig 2D and S1B Fig), which allowed the determination of NO levels in a physiological environment. These data showed that HTDAF-2 was a highly sensitive and selective fluorescent probe for NO, and it could be used for subsequent experiments in living cells.

### Subcellular detection of NO using HTDAF-2DA

**Fig 3:**
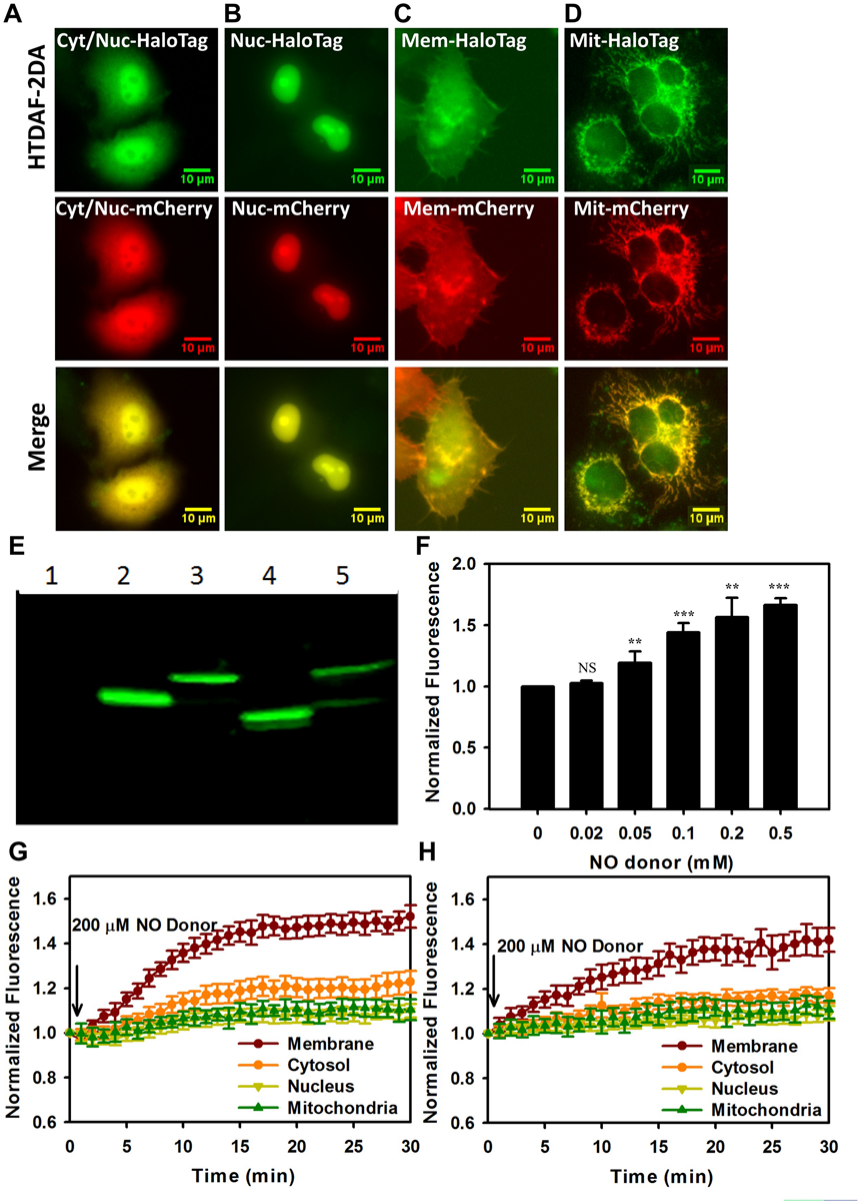
Fluorescence detection of NO in subcellular organelles of HeLa and MCF-7 cells. Targeted localization of HTDAF-2 by conjugation to HaloTag proteins in living HeLa cells. Images present HeLa cells expressing HaloTag in the cytosol/nucleus (A), nucleus (B), membrane (C), and mitochondria (D), with the red fluorescent protein mCherry fused with the same signal peptides. Scale bar = 10 μm. (E) Direct in-gel fluorescence of control (1), nucleus-HaloTag (2), plasma membrane-HaloTag (3), cytosol-HaloTag (4), and mitochondria-HaloTag (5) in HeLa cells labeled with 5 μM HTDAF-2DA. (F) The fluorescence responses of 5 μM HTDAF-2DA targeted in the plasma membrane to various concentrations of NO donor (DEA NONOate) in HeLa cells. (G and H) Kinetics of fluorescence response of 5 μM HTDAF-2DA in different subcellular compartments of HeLa (G) and MCF-7 (H) cells upon the addition of NO donor (DEA NONOate). Error bars represent SD.

In HeLa (cervical cancer cell line) cells, we targeted HaloTag to various subcellular compartments by tagging the protein with or without organelle-specific signal peptides, including the plasma membrane [27–29], cytosol [20–23], nucleus [24–26], and mitochondria [30–33]. After exposing the cells to HTDAF-2DA, washing away the unbound dye, and observing by fluorescent microscopy, we found that the HTDAF-2DA-labeled HaloTag protein produced excellent subcellular localization, which colocalized well with the red fluorescent protein mCherry fused with the same signal peptides (Fig 3A–3D). The correct localizations of HTDAF-2DA-labeled HaloTag protein can also be visualized in fluorescent microscopy images of cells co-stained with the blue fluorescent DNA staining dye DAPI or MitoTracker Red FM (S2A–S2D Fig). By contrast, DAF-2DA probe showed a low spatial resolution (S2E Fig). These data demonstrated that HTDAF-2DA was cell-permeable and could be applied to label specific organelles in living cells. We further analyzed the lysate of organelle HTDAF-2DA-stained cells with or without targeted HaloTag overexpression, by using denatured PAGE. A highly fluorescent band was observed only in HaloTag-overexpressed cells (Fig 3E), which indicated that HTDAF-2DA effectively, specifically, and covalently labeled the HaloTag protein in different subcellular compartments. Compared with other affinity-based tags, covalent labeling provides several advantages, including extending the labeling periods without dissociation of the label, imaging in chemically fixed cells, tolerating stringent wash conditions by immobilizing HaloTag on solid supports, and allowing multiplexing with immunocytochemical methods, SDS-PAGE, and western blot analysis [15, 35].

NO and NO synthases are ubiquitous in malignant tumors and are known to exert pro- and anti-tumor effects [3, 36, 37]. To understand NO responses in cancer biology, we analyzed HTDAF-2 fluorescence in different subcellular compartments of HeLa and MCF-7 (breast cancer cell lines). The results showed that exogenous NO addition led to the largest fluorescence changes in the membrane, followed by those in the cytosol, nucleus, and mitochondria (Fig 3F–3H). These results demonstrated that cell-permeable HTDAF-2DA could be used for in situ imaging of NO in living cells and tracing NO changes in different subcellular organelles. Moreover, the cell membrane was highly enriched with NO species.

**Fig 4:**
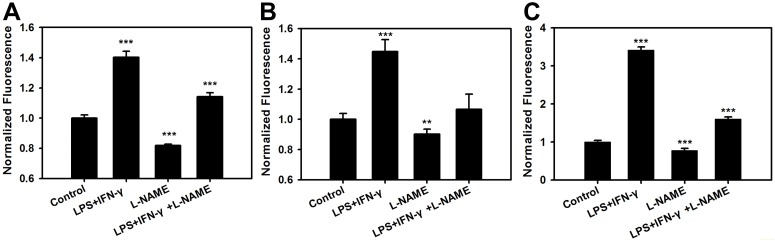
Measurement of endogenous NO production in activated macrophages by HTDAF-2DA and DAF-2DA. (A and B) NO detection in Raw 264.7 macrophages expressing HaloTag in the cytosol/nucleus (A) or nucleus (B) stained by HTDAF-2DA. (C) NO detection in Raw 264.7 macrophages stainedby DAF-2DA. For A-C, cells were prestimulated for 8 h with LPS (0.5 μg/ml) and IFN-γ (250 U/ml) with or without L-NAME (2 mM). Data were measured in pooled cells with microplate reader. Error bars represent SD.

It is well known that NO is produced by induced nitric oxide synthase in stimulated macrophages, which was monitored by using the Griess assay or NO fluorescent probes [6, 38–40]. However, these methods did not reveal intracellular NO production with spatial resolution. HTDAF-2DA, targeted to cytosol/nucleus or nucleus alone, readily detects subcellular NO produced in Raw 264.7 murine macrophages prestimulated with LPS and IFN-γ (Fig 4A and 4B). Furthermore, L-NAME, a known inhibitor of nitric oxide synthase, significantly decreased the increase of HTDAF-2DA fluorescence in LPS- and IFN-γ-treated macrophages, in agreement with previous reports [38–40]. Similar results were obtained when the production of NO was monitored using the widely used NO probe DAF-2DA (Fig 4C).

Direct determination of the spatiotemporal distribution of NO in situ is considerably significant in the exact function of NO in physiological and pathological conditions. However, convincing methods that provide spatiotemporal information of NO signaling are not yet available. Srikun et al. reported some organelle-targetable fluorescent probes for imaging hydrogen peroxide in living cells via SNAP-Tag protein labelling [41]. In this paper, we report a new organelle-targetable NO probe via HaloTag protein labeling. The presented methodology could also be extended to other small-molecule probes with genetically encoded protein scaffolds (e.g., HaloTag, SNAP-tag, and CLIP-tag).

In summary, we have described the design, synthesis, spectroscopic properties, NO responses, and subcellular NO imaging applications of a HaloTag-based fluorescent probe called HTDAF-2DA. This hybrid small-molecule/protein reporter could be used to label various subcellular compartments, including the plasma membrane, cytosol, nucleus, and mitochondria, as well as measure changes in local NO fluxes in living cells by microscopy. Several studies have demonstrated that high levels of NO can trigger cancer cell death via DNA damage, oxidative/nitrosative stress, cytotoxicity, and apoptosis [36, 42]. Various approaches for cancer treatment have been investigated, including NO-releasing drugs and NO as chemotherapy and radiotherapy sensitizers. Our results show that exogenous NO mainly accumulated in cancer cell membrane, which suggests that NO-triggered membrane damage may be the vanguard event of NO-induced cell death. The capability to image the spatial distribution of subcellular NO real time could be useful to better understand cellular signaling involving NO, including guanylyl cyclase activation, vessel homeostasis, neurotransmission, ischemia, inflammation, and neurodegeneration [43, 44]. HTDAF-2DA may also serve as a valuable tool for high-throughput screening of NO-donating drugs in cancer therapy. Further studies on expanding the color palette of targetable NO probes, optimizing the sensitivities and dynamic range of NO probes, and creating targetable sensors with ratiometric read-out are currently in progress.

## Supporting Information

S1 Fig Fluorescent properties of NO sensor HTDAF-2. (A) Comparison of the fluorescence intensities of HTDAF-2DA, HTDAF-2, HTdiAcFAM, and HTFAM. (B) Relative fluorescence intensities of HTDAF-2 with excitation at 485 nm and emission at 528 nm at the indicated pH. Data were normalized to the fluorescence at pH 7.4. Error bars represent SD.
(TIF)

S2 Fig HTDAF-2DA fluorescence images in HeLa cells expressing HaloTag and DAF-2DA fluorescence images. (A) The fluorescent microscopy images of HeLa cells expressing HaloTag in mitochondria co-stained with the blue fluorescent DNA staining dye DAPI or MitoTracker Red FM. (B-D) Images present HeLa cells expressing HaloTag in the cytosol (B), membrane (C), and nucleus (D) co-stained with DAPI. Scale bar = 10 μM. (E) DAF-2DA fluorescence images in HeLa cells. Scale bar = 10 μM.
(TIF)

S1 File Scheme A. Synthesis of HTDAF-2DA and HTDAF-2; Scheme B. Synthesis of HTdiAcFAM and HTFAM. (DOCX)
