# Supplementary figures and images for: Organelle-Specific Nitric Oxide Detection in Living Cells via HaloTag Protein Labeling
Source: PLoS One. 2015 Apr 29;10(4):e0123986. doi: 10.1371/journal.pone.0123986 (PMC4414533; doi:10.1371/journal.pone.0123986)

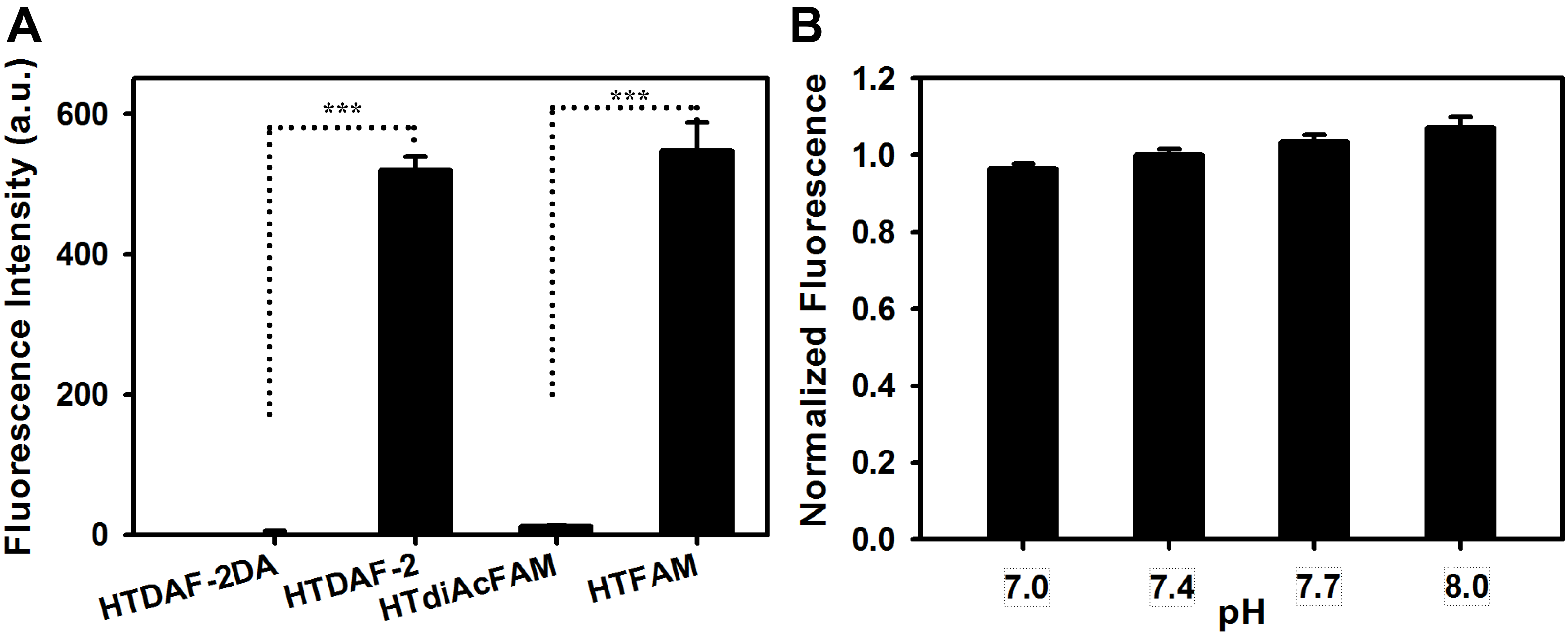

Supplement: S1 Fig — (A) Comparison of the fluorescence intensities of HTDAF-2DA, HTDAF-2, HTdiAcFAM, and HTFAM. (B) Relative fluorescence intensities of HTDAF-2 with excitation at 485 nm and emission at 528 nm at the indicated pH. Data were normalized to the fluorescence at pH 7.4. Error bars represent SD. (TIF) [file pone.0123986.s001.tif]

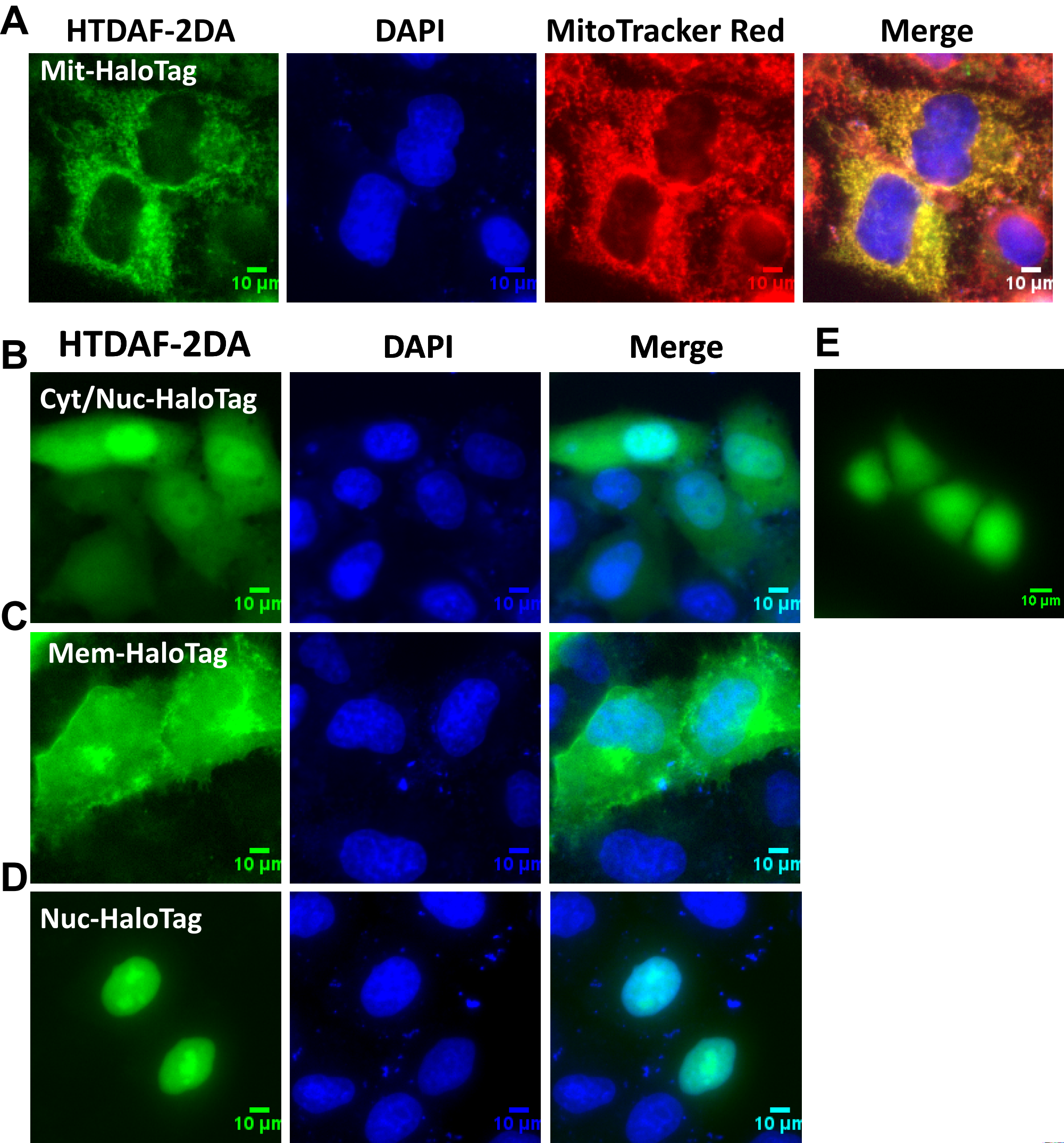

Supplement: S2 Fig — (A) The fluorescent microscopy images of HeLa cells expressing HaloTag in mitochondria co-stained with the blue fluorescent DNA staining dye DAPI or MitoTracker Red FM. (B-D) Images present HeLa cells expressing HaloTag in the cytosol (B), membrane (C), and nucleus (D) co-stained with DAPI. Scale bar = 10 μM. (E) DAF-2DA fluorescence images in HeLa cells. Scale bar = 10 μM. (TIF) [file pone.0123986.s002.tif]
