# Supplementary material for: Organelle-Specific Nitric Oxide Detection in Living Cells via HaloTag Protein Labeling
Source: PLoS One. 2015 Apr 29;10(4):e0123986. doi: 10.1371/journal.pone.0123986 (PMC4414533; doi:10.1371/journal.pone.0123986)
Supplement: S1 File — (DOCX) [file pone.0123986.s003.docx]

**Supporting information**

**Organelle-specific nitric oxide detection in living cells via** **HaloTag protein labeling**

Jianhua Wang ^1¶^, Yuzheng Zhao ^1¶^, Chao Wang ^2¶^, Qian Zhu^1^, Zengmin Du^1^, Aiguo Hu ^2^*, Yi Yang ^1^*

1 Synthetic Biology and Biotechnology Laboratory, State Key Laboratory of Bioreactor Engineering, School of pharmacy, East China University of Science and Technology, 130 Mei Long Road, Shanghai 200237, P. R. China.

2 Shanghai Key Laboratory of Advanced Polymeric Materials, School of Materials Science and Engineering, East China University of Science and Technology, 130 Mei long Road, Shanghai 200237, P. R. China.

* Corresponding author

E-mail: [yiyang@ecust.edu.cn(Yi](mailto:yiyang@ecust.edu.cn(Yi) Yang); [hagmhsn@ecust.edu.cn](mailto:hagmhsn@ecust.edu.cn)(Aiguo Hu)

^¶^ These authors contributed equally to this work.

**Experimental details**

**Characterization**

^1^H NMR and ^13^C NMR spectra were recorded in chloroform-d (CDCl_3_), CD_3_OD or DMSO-d6 on an Ultra Shield 400 spectrometer (Bruker BioSpin AG, Magnet System 400 MHz/54 mm). Mass spectra were generated with Micromass LCT (ESI-TOF). HPLC analysis was performed on a reversed-phase C18 column (5 μm, Agilent Eclipse XDB-C18, 4.6 mm × 150 mm). Products were detected by UV absorption at 280 nm, and fitted on an Agilent 1200 Series at a flow rate of 0.8 mL/min. Preparative HPLC purification was performed on a reversed-phase C18 column (10 μm, XB-C18, 10 mm × 150 mm). Products were detected by UV absorption at 280 nm, and fitted on a DIONEX UltiMate 3000 system at a flow rate of 3.0 mL/min. The mobile phase consisted of a mixture of H_2_O with 0.1% TFA (A) and CH_3_CN (B). The gradient started at 30% B, and was raised to 45% B within 5 min. The percentage of B increased linearly from 5 min to 60% at 45 min.

**Synthetic routes and details**

**2-bromo-*N*-{2-[2-(6-chloro-hexyloxy)-ethoxy]-ethyl}-acetamide (2)**

Potassium carbonate (1.66 g, 12 mmol) was added to a solution of 2-[2-(6-chloro-hexyloxy)-ethoxy]-ethylammonium hydrochloride (1) (1.04 g, 4 mmol) in CH_2_Cl_2_ (12 mL), and the resulting mixture was stirred in an ice bath. Bromoacetyl bromide (0.78 mL, 9 mmol) in CH_2_Cl_2_ (6 mL) was added dropwise over a period of 30 min. The reaction mixture was stirred for 5 h in an ice bath, and quenched with water. The organic extract was washed with HCl (10%) and then with water, dried over MgSO_4_, and filtered. After solvent removal, the residue was purified by column chromatography over silica gel eluting with PE/EtOAc (2:1, v/v) to obtain a pale yellow oil (1.03 g, 75%). ^1^H NMR (CDCl_3_) δ: 1.40 (m, 4H, -CH_2_-), 1.60 (m, 2H, -CH_2_-), 1.77 (m, 2H, -CH_2_-), 3.40-3.70 (m, 12H, CH_2_-N, CH_2_-O, CH_2_-Cl), 3.85 (s, 2H, CH_2_-Br), 6.99 (s, 1H, NH) ppm. ^13^C NMR (CDCl_3_) δ: 25.4, 26.7, 29.1, 29.4, 32.5, 39.9, 45.1, 69.4, 70.0, 70.4, 71.3, 165.7 ppm. HRMS: m/z calculated for M + Na 366.0448, found 366.0445.

**HTDAF-2**

HTDAF-2DA (13.4 mg, 0.019 mmol) was dissolved in MeOH (2 mL) and H_2_O (1.5 mL), and 30% aqueous NH_4_OH (1.3 mL) was added. The mixture was stirred at room temperature for 4 h. The solvent was also removed in vacuo at low temperature to obtain a quantitative yield of HTDAF-2. ^1^H NMR (DMSO-d6) δ: 1.46 (m, 4H, -CH_2_-), 1.68 (m, 2H, -CH_2_-), 1.98 (m, 2H, -CH_2_-), 3.42-3.66 (m, 14H, CH_2_-N, CH_2_-O, CH_2_-Cl), 6.55 (dd, 2H, ArH, *J* = 8.4, 2.4 Hz), 6.66 (d, 2H, ArH, *J* = 8.4 Hz), 6.70 (d, 2H, *J* = 2.4 Hz) 6.89 (s, 1H, ArH), 8.33 (s, 1H, ArH), 10.23 (s, 2H, OH) ppm.

**HTFAM**

2-[2-(6-Chloro-hexyloxy)-ethoxy]-ethylammonium hydrochloride (1) (77 mg, 0.30 mmol), *N*-(3-dimethylaminopropyl)-N′-ethylcarbodiimide hydrochloride (EDC·HCl) (62 mg, 0.32 mmol), and diisoproplyethylamine (0.5 mL, 2.9 mmol) were added to 2 mL of dry DMF. 6-Carboxyfluorescein (101 mg, 0.27 mmol) and 4-dimethylaminopyridine (4.9 mg, 0.04 mmol) were then added to the mixture. The mixture was stirred under nitrogen at room temperature for 24 h. The solvent was removed in vacuo. The residue was purified by column chromatography over silica gel to yield an orange solid (58 mg, 37%). ^1^H NMR (CD_3_OD) δ: 1.40 (m, 4H, -CH_2_-), 1.59 (m, 2H, -CH_2_-), 1.79 (m, 2H, -CH_2_-), 3.40-3.70 (m, 12H, CH_2_-N, CH_2_-O, CH_2_-Cl), 6.54 (dd, 2H, *J*= 8.8, 2.4 Hz), 6.69 (d, 2H, *J*= 2.4 Hz), 6.83 (d, 2H, *J*= 8.4 Hz), 7.30 (d, 1H, *J*= 7.6 Hz), 7.81 (dd, 1H, *J*= 8.0, 1.2 Hz), 8.05 (s, 1H) ppm.

**HTdiAcFAM**

HTFAM (41 mg, 0.070 mmol) and Cs_2_CO_3_ (27 mg, 0.083 mmol) were added to 5 mL of dry acetonitrile, and acetic anhydride (56 μL, 0.169 mmol) was added dropwise. The mixture was stirred at room temperature for 2 h, and then filtered. The solvent was removed in vacuo. The residue was purified by column chromatography over silica gel to yield a pale yellow solid (19 mg, 41%). ^1^H NMR (CDCl_3_) δ: 1.40 (m, 4H, -CH_2_-), 1.60 (m, 2H, -CH_2_-), 1.75 (m, 2H, -CH_2_-), 2.31 (s, 6H, COCH_3_), 3.40-3.70 (m, 12H, CH_2_-N, CH_2_-O, CH_2_-Cl), 6.54 (dd, 2H, *J*= 8.0, 2.4 Hz), 6.74 (d, 2H, *J*= 2.0 Hz), 6.82 (d, 2H, *J*= 8.4 Hz), 7.08 (s, 1H), 7.30 (d, 1H, *J*= 7.6 Hz), 7.74 (dd, 1H, *J*= 8.0, 1.2 Hz) ppm.

**Supplementary Scheme**


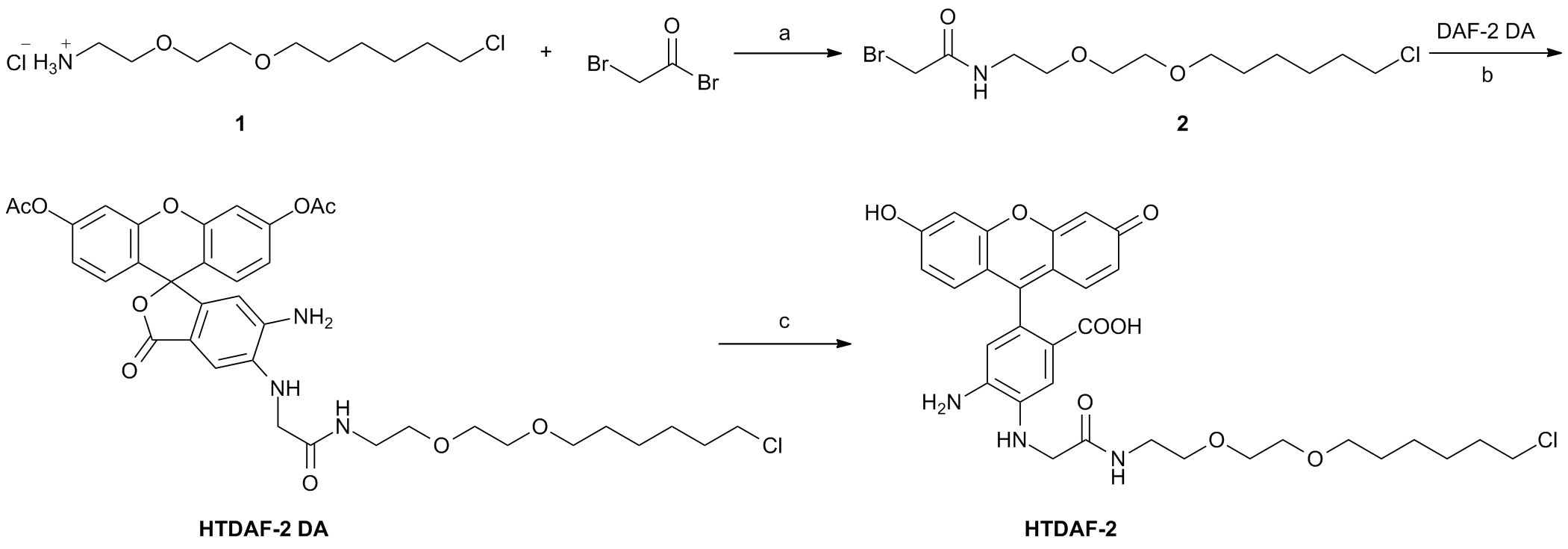


**Scheme A. Synthesis of HTDAF-2DA and HTDAF-2.** a: K_2_CO_3_, CH_2_Cl_2_, 0 °C, 5 hr; b: K_2_CO_3_, NaI, DMF, r.t., 24hr; c: 30% NH_4_OH (aq.), MeOH/H_2_O, r.t., 4 hr.

**
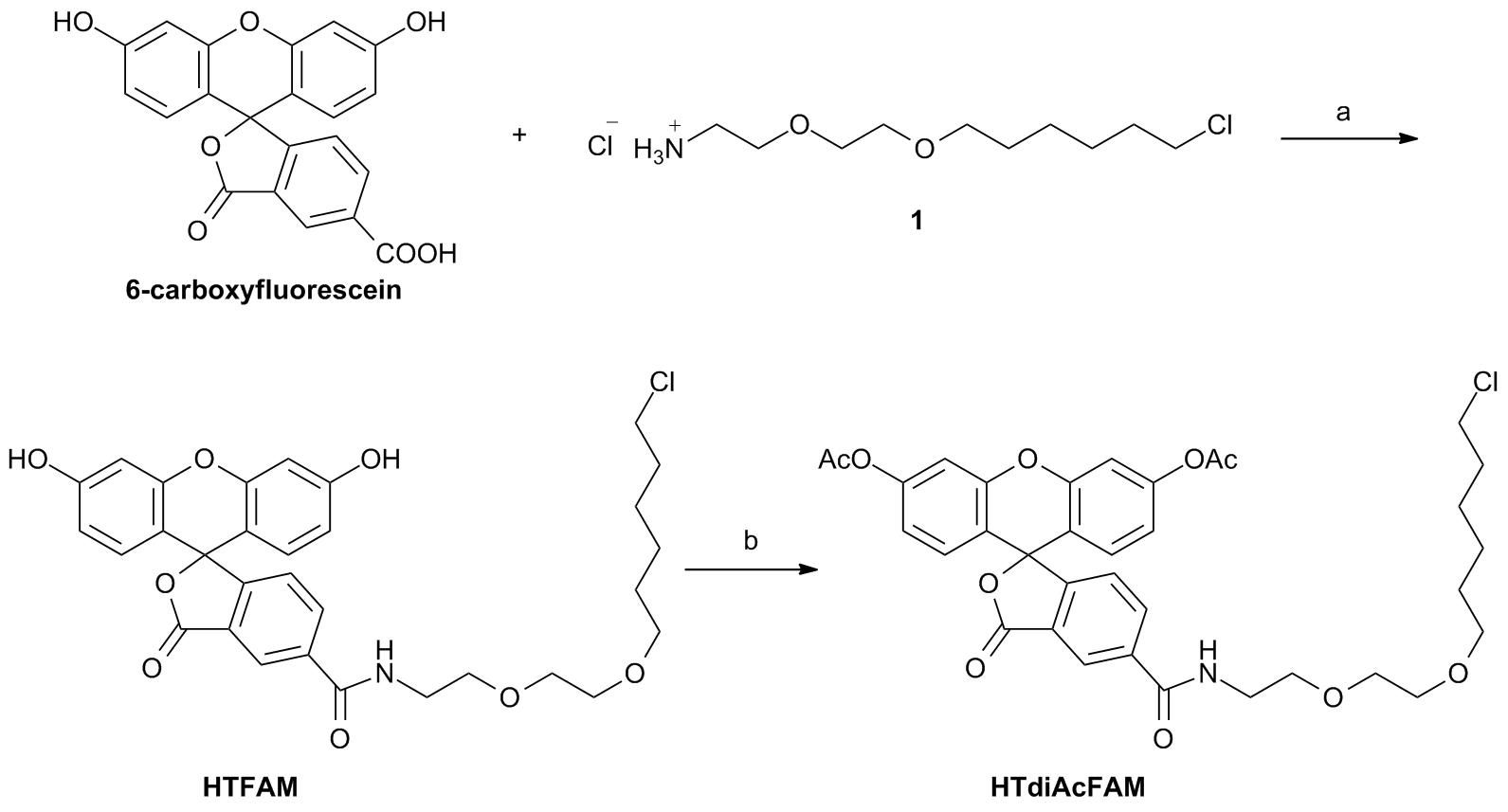
**

**Scheme B. Synthesis of HTdiAcFAM and HTFAM.** a: EDC, DMAP, DIPEA, DMF, r.t. 24 hr; b: Ac_2_O, Cs_2_CO_3_, CH_3_CN, r.t. 2 hr.

**S1** **Fig. Fluorescent properties of NO sensor HTDAF-2.** (A) Comparison of the fluorescence intensities of HTDAF-2DA, HTDAF-2, HTdiAcFAM, and HTFAM. (B) Relative fluorescence intensities of HTDAF-2 with excitation at 485 nm and emission at 528 nm at the indicated pH. Data were normalized to the fluorescence at pH 7.4.

Error bars represent SD.

**S2 Fig. HTDAF-2DA ﬂuorescence images in Hela cells expressing HaloTag and DAF-2DA ﬂuorescence images.** (A) The fluorescent microscopy images of Hela cells expressing HaloTag in mitochondria co-stained with the blue fluorescent DNA staining dye DAPI or MitoTracker Red FM. (B-D) Images present HeLa cells expressing HaloTag in the cytosol/nucleus (B), membrane (C), and nucleus (D) co-stained with DAPI. Scale bar = 10 µM. (E) DAF-2DA ﬂuorescence images in HeLa cells. Scale bar = 10 µM.
